# UNNECESSARY AND HARMFUL MEDICATION USE IN COMMUNITY DWELLING PERSONS WITH DEMENTIA

**DOI:** 10.1093/geroni/igac059.1626

**Published:** 2022-12-20

**Authors:** W James Deardorff, Bocheng Jing, Matthew Growdon, Kristine Yaffe, Kenneth Boockvar, Michael Steinman

**Affiliations:** University of California, San Francisco, San Francisco, California, United States; University of California, San Francisco, San Francisco, California, United States; University of California, San Francisco, San Francisco, California, United States; University of California, San Francisco, San Francisco, California, United States; Icahn School of Medicine at Mount Sinai, New York, New York, United States; University of California, San Francisco, San Francisco, California, United States

## Abstract

Persons with dementia (PWD) often have multiple comorbidities which results in extensive medication use despite potentially limited benefit and increased risk of adverse events. Compared to the nursing home, little is known about medication overuse and misuse among the ~70% of PWD in the community. Therefore, we examined medication use from Medicare Part D prescriptions among 1,289 community-dwelling PWD aged ≥66 from the Health and Retirement Study. We classified medication overuse as over-aggressive treatment of chronic conditions (e.g., insulin/sulfonylurea use with hemoglobin A1c<7.5%) and medications inappropriate near the end of life. We classified medication misuse as medications that negatively affect cognition (strongly anticholinergics/sedative-hypnotics) and problematic medications (using Beers and STOPP criteria). We describe the prevalence and patterns of different types of medication overuse/misuse. Frequently problematic medications included antipsychotics (9%), benzodiazepines (12%), and gabapentinoids (13%). Our findings highlight the burden of unnecessary/harmful medications among PWD and inform future deprescribing interventions.

